# Indirect Orbital Floor Fractures: A Meta-Analysis

**DOI:** 10.4103/0974-9233.63076

**Published:** 2010

**Authors:** Mithra O. Gonzalez, Vikram D. Durairaj

**Affiliations:** Department of Ophthalmology, Division of Oculoplastic and Orbital Surgery, Rocky Mountain Lions Eye Institute, University of Colorado, Aurora, CO, USA

**Keywords:** Blowout, Diplopia, Evaluation, Fracture, Management, Orbital floor, Trauma

## Abstract

Orbit fractures are common in the context of orbital trauma. Fractures of the orbital floor without orbital rim involvement are known as indirect orbital floor fractures, pure internal floor fractures, and orbital blowout fractures. In this paper, we have reported a meta-analysis of orbital floor fractures focusing on indications and timing of surgical repair, outcomes, and complications.

## INTRODUCTION

Orbital fractures are common in facial and orbital trauma, with orbital floor and medial wall commonly affected. Fractures may be either direct or indirect. Direct orbital fractures involve and extend from the orbital rim. Indirect orbital floor fractures are therefore fractures of the orbital floor without involvement of the orbital rim. This fracture configuration has been described by several names including indirect orbital floor fracture, blowout fracture, and pure internal floor fracture. Two proposed mechanisms are employed to explain orbital floor fractures with an intact orbital rim. Smith and Regan proposed that compression of the globe increases the hydraulic pressure; it exerts on the orbital floor, which if sufficient, results in a fracture.^1^,^2^ Fujino postulated that forces applied to the inferior orbital rim are transmitted posteriorly through the thin orbital floor and may result in a buckling of bone and thus produces an indirect orbital floor fracture.^3^

## INDICATIONS AND TIMING OF SURGICAL REPAIR

Although indications for repair of indirect orbital floor fractures vary by specialty, be it otolaryngology, maxillofacial, or plastic surgery, more studies have been published within the ophthalmic literature and consistent recommendations have been generated. The contents of this review have been primarily derived from the ophthalmic literature. Burnstine reported an evidenced-based analysis on the management of orbital floor fractures.^4^ The analysis graded available current recommendations in two dimensions. First, the “importance to care” was judged using a level system, i.e., levels A, B, or C. Level A was defined as the most important to clinical outcome. Level B was defined as moderately important to clinical outcome. Level C was defined as relevant, but not critical to clinical outcome. Second, the strength of evidence as determined by available literature for a given recommendation and was also judged using a level system, i.e., levels I, II, or III. Level I was defined as data that provided strong supporting evidence of a given recommendation. Level II was defined as data that provided substantial evidence for a given recommendation. Level III was defined as data that lacked or had weak evidence to support or refute a given recommendation.

**Figure 1:**
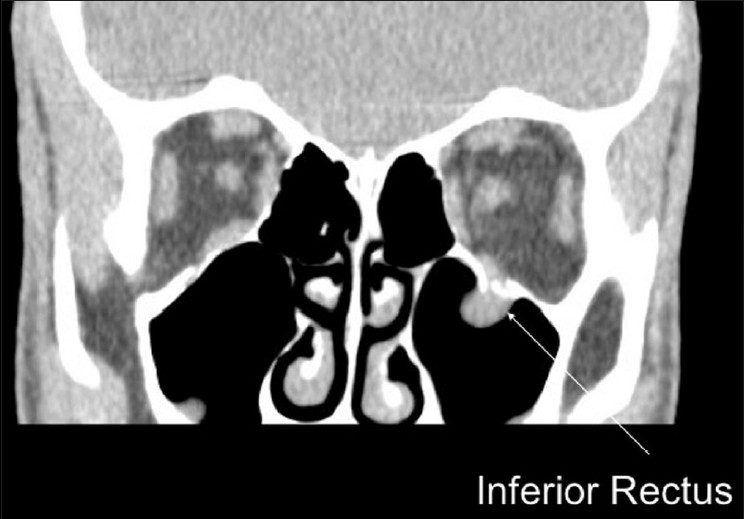
Left orbital floor fracture with entrapment of inferior rectus

**Figure 2:**
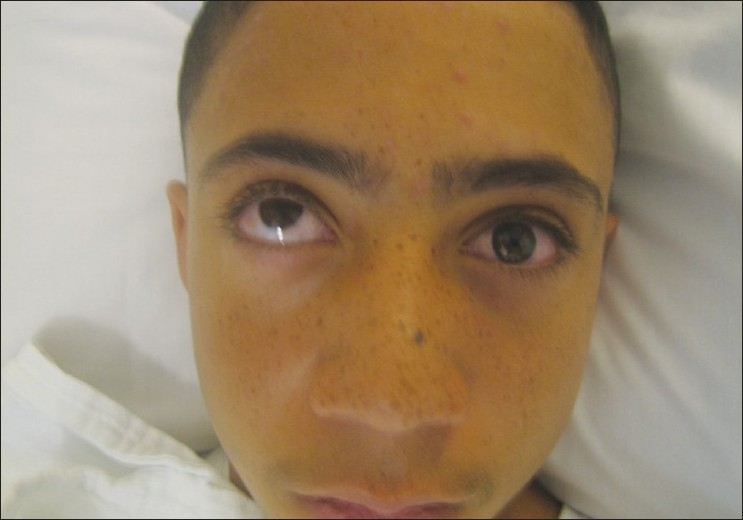
Left orbital floor fracture with severe restriction in left upgaze secondary to entrapment of inferior rectus

**Figure 3:**
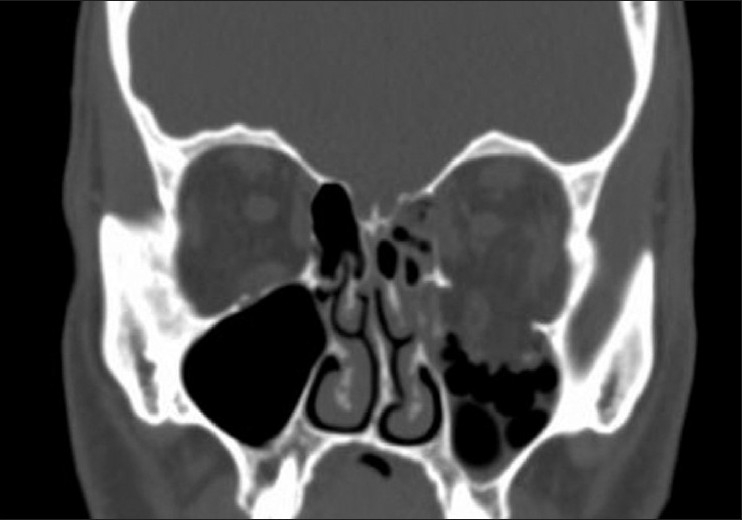
Large left orbital floor fracture greater than 50% of orbital floor

Immediate repair (within 24–48 h) is recommended with AI level evidence for diplopia present with computed tomography (CT scan) evidence of muscle or soft tissue entrapment and a nonresolving oculocardiac reflex [Figure 1]. Immediate repair is also recommended for “white-eyed blowout fractures,” in patients less than 18 years of age, with a history of periocular trauma, minimal ecchymosis or edema, marked extraocular motility vertical restriction, and CT-scan findings of an orbital floor fracture with muscle or peri-muscular soft tissue entrapment^4^,^5^ [Figure 2]. Early repair within 2 weeks is recommended with varying levels of evidence for a variety of clinical settings. The repair of orbital floor fractures with symptomatic diplopia with positive forced ductions and CT-scan documentation of inferior rectus muscle or peri-muscular soft tissue entrapment and minimal clinical improvement, is recommended within 2 weeks as is repair of fractures that result in significant hypoglobus with level A:II evidence. Within 2 weeks, repair is recommended for large floor fractures (typically greater than 50% of floor) causing latent enophthalmos with level B:II evidence [Figure 3]. Early repair of floor fractures is recommended for progressive infraorbital hypoesthesia with level C:III quality evidence. Observation is recommended if there is minimal diplopia that is not present in primary or down-gaze, good ocular motility, and no significant enophthalmos or hypoglobus.^4^,^5^

Lelli *et al*. have provided an excellent review of ophthalmic indications and approaches to repair of orbital floor fractures.^6^ They describe two emergent conditions that require urgent surgical repair: (1) diplopia with radiological evidence of muscle or soft tissue entrapment and a nonresolving oculocardiac reflex, and (2) “white-eyed” fractures in patients less than 18 years of age with vertical limitation of eye movement and radiological evidence of inferior rectus muscle or peri-muscular soft tissue entrapment. Otherwise, subacute surgical repair is advised to take place “as soon as reasonably possible,” once surgery has been deemed necessary. Indications for subacute repair include: clinical evidence of enophthalmos or hypoglobus that is functionally or cosmetically concerning, diplopia in the setting of either positive forced duction testing or radiological evidence of entrapment, or extensive floor fractures (>50% of the orbital floor). Individuals with diplopia, limited versions, but without radiological evidence of entrapment, should be monitored closely (5–7 days after the injury), and improvement should be observed in 1–2 weeks.^6^ If the motility dysfunction does not improve or stabilize, surgery should be considered.^6^ Cases of minimal diplopia and especially diplopia that does not affect primary or down-gaze, with small fractures and good motility may be largely observed. Exceptions to this scenario include athletes, electricians, or others whose occupation requires singular vision in upgaze.

## OUTCOMES

The timing of repair of indirect orbital floor fractures has been the subject of numerous reports. Putterman *et al*. observed 57 patients with orbital floor fractures, two-thirds of whom presented with primary gaze diplopia and limited vertical ductions, and found that at the time of their last examination, nearly in all patients diplopia had resolved in primary position for nearly all patients.^7^ Hawes and Dortzbach noted that postoperative enophthalmos was minimized if floor defects measuring >50% were repaired within 2 weeks.^8^ Fat atrophy and scarring were the hypothesized cause of this phenomenon. Dal Canto and Linberg found that patients faired equally if their orbital floor fractures were repaired within 14 days or within 29 days after trauma.^9^ In their retrospective study of 58 patients, 36 had early repair within 14 days (mean of 9 days) compared to 22 patients who had delayed repair (mean of 19 days). The end results showed no statistical difference in postoperative binocular diplopia when comparison of early versus late fracture repair was carried out. In primary gaze, 8.3% of individuals of the early repair group had postoperative binocular diplopia compared to 4.6% reported in the delayed repair group.^9^ In reading gaze, 16.7% of the early repair group reported postoperative binocular diplopia as compared to 9.1% in the delayed repair group. In other gazes, early repair was associated with 41.7% binocular diplopia as compared to 45.5% in the delayed repair group. There was no statistical difference in the need for postoperative strabismus surgery or prisms when comparing early versus delayed repair.^9^ However, the authors did report that delayed repair was more technically challenging and that when surgery is a forgone conclusion, early repair (within 1–2 weeks) is preferable. Furthermore, the authors specifically stated that their results do not apply to “white-eyed blowout fractures,” and although unstated, nor to those with a nonresolving oculocardiac reflex.^9^

Recently, Simon *et al.* reported that postoperative outcomes were similar between those patients with indirect orbital floor fractures who had early repair when compared to those with late repair.^10^ They carried out surgery if there was evidence for the presence of inferior rectus muscle entrapment or limitation in upgaze (40% of surgeries), or anticipated enophthalmos (60% of surgeries). Early repair was performed with the first 2 weeks. Late repair was performed 1 month to 3.5 years after the injury. No average time was given for late repair group. Of the 50 consecutive patients with orbital fractures, 34 (68%) had evidence of isolated orbital floor fractures. Preoperative enophthalmos was seen in 34 (68%) of patients, 20 (40%) of whom had 2 mm or greater enophthalmos.^10^ Given the comparable postoperative results of indirect orbital floor fracture repair, immediate intervention was recommended for cases of diplopia with imaging evidence of muscle or peri-orbital entrapment, “white-eyed blowout fractures,” and those with a nonresolving oculocardiac reflex.^9^,^12^ Early intervention (within 2 weeks) was recommended for those with symptomatic diplopia and positive forced-duction testing with the evidence of entrapment as well as those with large floor fractures that would result in significant enophthalmos.^9^,^12^ Simon *et al*. recommendations concur with reports made by other clinicians.^4^,^5^ However, their findings did provide additional insight into the clinical course of subset of orbital floor fractures. Specifically in adults, duction limitations is not caused by obvious muscle entrapment, but rather injury to the muscle or soft tissue, which may resolve over 6–9 months without surgical repair.^10^ Similar postoperative outcomes of early versus late repair of orbital floor fractures allow for an extended “watchful waiting” period in cases without inferior rectus muscle entrapment and perhaps in a select group of patients, entirely obviates the need for surgery.

## PEDIATRIC INDIRECT ORBITAL FRACTURES

Pediatric indirect orbital floor fractures deserve special attention as they can behave differently than their adult counterparts. “White-eyed,” orbital floor blowout fractures are typically found in children. Originally described by Jordon *et al*., these fractures present with a history of periorbital trauma, minimal ecchymosis, or erythema, profound vertical motility restriction and minimal radiological findings.^11^ Perhaps because of the tendency to form trapdoor fractures that entrap the inferior rectus muscle or its peri-muscular soft tissue, this subset of orbital floor fractures often require urgent repair. Their analysis found that repair should be performed within the first few days after injury and that delay may be to the detriment of postoperative motility.^11^ Trapdoor fractures were further characterized by Bansagi and Meyer. In their retrospective observational case series of 34 pediatric patients between the age group 1 and 18 years with indirect orbital fractures.^11^ Of the 34 patients, 59% had indirect orbital floor fractures. In patients with orbital floor fractures, 13 were noted to have impaired supraduction whereas 11 had impaired infraduction. Patients with trapdoor fractures (88% of trapdoor fractures affecting the orbital floor) were statistically more likely to have nausea/vomiting and motility restriction, with a predictive value of nausea and vomiting for identifying trapdoor at 71%. The authors noted that prompt repair may prevent muscle ischemia and potentially fatal arrhythmias secondary to the oculocardiac reflex.

Egbert *et al.*^12^ performed a noncomparative, retrospective, analysis on a consecutive case series of 34 patients less than 18 years of age with isolated orbital floor fractures to determine their clinical presentation, operative findings, and postoperative results. Presenting signs and symptoms in their patients included severe limitation of ductions in 26 of 34 (76%) subjects, pain with extraocular movement in 18 of 34 (53%) subjects, enophthalmos in 11of 34 (32%) subjects, and nausea/vomiting in 9 of 34 (26%) subjects. CT scan of these patients showed clear evidence of inferior rectus muscle entrapment in 18 of 34 (53%) subjects. Trapdoor fractures were most common which were present in 21 of 34 (62%) subjects followed by large fractures in 26% of subjects, hinged fractures in 9% of subjects and comminuted fractures in 3% of subjects. The relative frequency of trapdoor fractures in children when compared to adults is possibility related to differences in bone age, i.e., immature versus mature bone, with mature bone being more likely to shatter and result in large comminuted fractures while immature bone is more likely to bend and break as greenstick fracture.^12^ Trapdoor fractures were statistically more likely to have severe limitations in 20 of 21 (95%) subjects compared to other types of fractures.^12^ In their series, repair was performed within 7 days of fracture more rapidly resolved diplopia and limitation of ductions when compared to those repaired 2–4 weeks after injury although no postoperative difference was present between those repaired within 7 days compared to those repaired within 1 month. In conclusion, Egbert *et al*. recommend early surgery for pediatric patients with severe duction deficits, muscle entrapment with particular sensitivity to those with nausea, vomiting, pain with eye movement, and of a nonresolving oculcardiac reflex.^12^

## COMPLICATIONS

Complications of indirect orbital floor fractures result from a loss in the structural integrity of the orbital floor and its sequelae such as redistribution of or injury to orbital content and from injury to tissue or imprecise correction during the repair of floor fractures. Orbital floor fractures, either direct or indirect, may result in diplopia, inferior rectus muscle or peri-muscular soft tissue entrapment with subsequent tissue ischemia, fibrosis or necrosis, mydriasis, hypoglobus, enophthalmos, V2 distribution pain or paresthesia, severe orbital emphysema, nausea/vomiting, or a nonresolving oculocardic reflex.^4^,^5^,^7^–^14^ Surgical complications can include but are not limited to optic nerve injury, blindness, vision loss, infection or migration of implanted material, pyogenic granuloma formation, postoperative mydriasis, epiphora, worsening diplopia, and lid malpositioning.^4^,^5^ Biesman *et al*. showed that 37% of patients with isolated floor fractures had persistent postoperative diplopia.^15^ When both an orbital floor and medial wall fracture were present, persistent postoperative diplopia was seen in 86% of patients.^15^ Lee and Nunery^16^ recently published a retrospective review of 10 consecutive patients who presented with diplopia and/or cicatricial eyelid retraction after repair of orbital fracture with titanium implants. Of 10 patients, 6 had cicatricial eyelid retraction, and 9 of 10 patients had extraocular motility restriction and resulting diplopia.^16^ Replacement of titanium with 0.4-mm nylon implants and repair of the cicatricial eyelid retraction by fibrolysis and full-thickness skin grafting improved all diplopia and improved eyelid position. One of the clinical virtues of titanium implants is their ability to integrate into native tissue and thus avoid extrusion. This ability to integrate and cause fibrosis may contribute to the complications found with titanium implants. To avoid orbital adherence syndrome, Lee and Nunery recommended using nonporous, nonreactive implants for intraorbital fracture repair, minimal eyelid dissection, and when titanium plating is necessary, using the smallest plate possible (1.0 mm low-profile plates) and placing the plates as far as possible away from the orbit and eyelid tissue.^16^
